# The heterogeneous herd: Drivers of close‐contact variation in African buffalo and implications for pathogen invasion

**DOI:** 10.1002/ece3.10447

**Published:** 2023-08-22

**Authors:** Julie Rushmore, Brianna R. Beechler, Hannah Tavalire, Erin E. Gorsich, Bryan Charleston, Anne Devan‐Song, Caroline K. Glidden, Anna E. Jolles

**Affiliations:** ^1^ Carlson College of Veterinary Medicine Oregon State University Corvallis Oregon USA; ^2^ One Health Institute, School of Veterinary Medicine University of California Davis California USA; ^3^ EpiCenter for Disease Dynamics, School of Veterinary Medicine University of California Davis California USA; ^4^ Department of Integrative Biology Oregon State University Corvallis Oregon USA; ^5^ The Zeeman Institute: Systems Biology and Infectious Disease Epidemiology Research University of Warwick Coventry UK; ^6^ School of Life Sciences University of Warwick Coventry UK; ^7^ The Pirbright Institute Guildford UK

**Keywords:** biological trait, individual reproduction number, proximity logging collar, social network analysis, *Syncerus caffer*

## Abstract

Many infectious pathogens are shared through social interactions, and examining host connectivity has offered valuable insights for understanding patterns of pathogen transmission across wildlife species. African buffalo are social ungulates and important reservoirs of directly‐transmitted pathogens that impact numerous wildlife and livestock species. Here, we analyzed African buffalo social networks to quantify variation in close contacts, examined drivers of contact heterogeneity, and investigated how the observed contact patterns affect pathogen invasion likelihoods for a wild social ungulate. We collected continuous association data using proximity collars and sampled host traits approximately every 2 months during a 15‐month study period in Kruger National Park, South Africa. Although the observed herd was well connected, with most individuals contacting each other during each bimonthly interval, our analyses revealed striking heterogeneity in close‐contact associations among herd members. Network analysis showed that individual connectivity was stable over time and that individual age, sex, reproductive status, and pairwise genetic relatedness were important predictors of buffalo connectivity. Calves were the most connected members of the herd, and adult males were the least connected. These findings highlight the role susceptible calves may play in the transmission of pathogens within the herd. We also demonstrate that, at time scales relevant to infectious pathogens found in nature, the observed level of connectivity affects pathogen invasion likelihoods for a wide range of infectious periods and transmissibilities. Ultimately, our study identifies key predictors of social connectivity in a social ungulate and illustrates how contact heterogeneity, even within a highly connected herd, can shape pathogen invasion likelihoods.

## Graphical abstract

Using proximity‐logging collars and network analyses, our study found considerable close‐contact heterogeneity in an African buffalo herd, with particular life‐history traits predicting association patterns. Through mathematical modeling, we demonstrated how the observed heterogeneity drives invasion likelihood at time scales relevant to infectious pathogens. Our study provides a framework that links host connectivity and infectious disease biology to characterize a population's pathogen invasion landscape and identify classes of superspreading hosts.

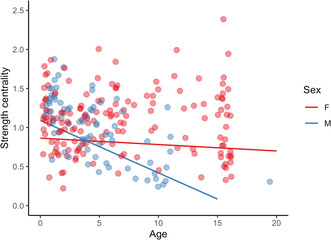

## 1 INTRODUCTION

The development of social structure and how individuals interact within populations is a foundational topic in animal behavior, with important implications for the transfer of information (McComb et al., 2001), genes (Altmann et al., 1996), and pathogens (Altizer et al., 2003). In particular, there is mounting evidence that a population's social structure can impact the invasion likelihood of a pathogen, the speed at which it spreads, and the number of individuals it infects (Keeling, 1999; Newman, 2002; Romano et al., 2020; Sah et al., 2018). Network analysis has become a powerful tool for examining linkages between wildlife social connectivity and disease, especially for primates, rodents, and reptiles (Godfrey, 2013; Rushmore et al., 2017; Sah et al., 2021; White et al., 2017). Social networks have been described for a handful of herd‐living species, offering valuable insights into social structure at the population level for ungulates (African buffalo: Cross et al., 2004; Onagers and Grevy's zebra: Sundaresan et al., 2007; reticulated giraffe: VanderWaal, Wang, et al., 2014; alpine ibex: Brambilla et al., 2022). These studies typically consider individuals to be associating if they are in the same group, which assumes that groups are well‐mixed. However, relatively little is known about the close‐contact patterns of herd‐living (rarely solitary) ungulate species and the resulting consequences for pathogen invasion.

Among social species, individuals typically exhibit considerable variation in contact rates, as demonstrated by social networks (dolphins: Lusseau, 2003; deer mice: Clay et al., 2009; spider monkeys: Rimbach et al., 2015; red deer: Albery et al., 2021). Many wildlife species have complex social structures in which relatedness and life history traits (e.g., age, sex, or reproductive status) play an important role in determining how frequently individuals interact with conspecifics. For example, in bighorn sheep, contact rates between lambs and reproductive ewes are orders of magnitude higher than contact rates among other group members (Manlove et al., 2017). Examining how individual traits affect contact heterogeneity can identify groups of individuals that play important roles in contact‐driven processes, such as pathogen transmission. Superspreaders are a common feature of infectious disease epidemics, where a small portion of well‐connected individuals are responsible for a majority of transmission events (Lloyd‐Smith et al., 2005). Such heterogeneities in individual transmission potentials can profoundly affect the course of an outbreak and strategies for disease control, underscoring the need to elucidate drivers of transmission heterogeneities (Lloyd‐Smith et al., 2005; Salathé et al., 2010; VanderWaal & Ezenwa, 2016).

Given their large and typically well‐connected populations, herd‐living ungulates frequently serve as reservoirs for infectious diseases that circulate among wildlife species and at the wildlife‐livestock interface (Barroso et al., 2021; Coetzer et al., 1994). African buffalo (*Syncerus caffer*) are considered the primary maintenance hosts for foot‐and‐mouth disease virus (FMDV: Bastos et al., 2000; Jolles et al., 2021; Vosloo et al., 2002) and a maintenance host for *Mycobacterium bovis* (causative agent of bovine tuberculosis: Jolles et al., 2005; Renwick et al., 2007) in African ecosystems. Buffalo are often implicated in transmission events that affect wildlife species and cattle farms, with profound effects on wildlife management and local human livelihoods (Bastos et al., 2000; Michel & Bengis, 2012; Omondi et al., 2020; Vosloo et al., 2002). Understanding how life history traits correspond to buffalo connectivity could help clarify how individual buffalo contribute to population‐level disease outbreaks.

African buffalo are social ungulates that live in large herds (*N* = 30–1500). Buffalo demonstrate nonrandom association patterns (Cross et al., 2005; Sinclair, 1977; Turner et al., 2005), which may translate into predictable differences in individual connectivity and infection risk. Specifically, buffalo have a fission‐fusion social structure, whereby individuals form separate groups that rejoin and mix over time (Cross et al., 2005; Prins, 1996), with adult dispersal occurring in both males and females (Spaan et al., 2019). The basic family unit consists of a mother and her one or two most recent calves (Sinclair, 1977). Calves are born in the wet season (November to April) after an 11‐month gestation period (Fairall, 1968; Ryan et al., 2006). They lose maternally derived immunity to some pathogens around 4–6 months of age (Jolles et al., 2021), but remain associated with their mothers for 1.5–2 years, often remaining close even after the next calf is born (Prins, 1996). Juveniles gradually spend more time away from their mothers within the herd, and by 4 years of age, males start to leave the breeding herd for bachelor groups during dry seasons (Turner et al., 2005). As they age, some adult males stop returning to the breeding herd and remain in small mature male bachelor groups (Prins, 1996; Sinclair, 1977). While broad‐scale social patterns are well documented in this species, little is known about associations at a finer scale.

Here, we analyzed temporally dynamic African buffalo social networks to gain insight into close association patterns among herd members, with a focus on identifying drivers of contact heterogeneity and pathogen invasion. Epidemiological models for gregarious ungulate herds have historically been limited by the assumption that herds are well mixed with respect to pathogen transmission. By combining detailed data on buffalo association patterns, life history traits, and genetics with models of disease spread, we were able to test this assumption and investigate how individual characteristics scale up to affect group‐level processes, with valuable insights for identifying key traits of potential superspreaders. First, we examined how genetic relatedness, age, sex, reproductive status, and measures of animal quality affect connectivity. Given that mothers and young calves remain as family units for up to 2 years in breeding herds, we expected genetic relatedness, age, and sex to be the most important predictors of buffalo association patterns, with males becoming less connected as they reach puberty and join bachelor herds. Next, we examined how the observed social network could affect a pathogen's potential ability to invade and spread within the herd, given a range of pathogen infectious periods and transmissibilities. This work provides insights into how data on contact patterns and life histories can reveal hidden heterogeneities capable of shaping pathogen invasion likelihoods, even in seemingly well‐mixed populations.

## 2 MATERIALS AND METHODS

### 2.1 Study site and population

Kruger National Park (KNP) spans nearly 19,485km^2^ (22.5°–25.5° S, 31.0°–31.57° E) and hosts a diversity of wildlife, including wild African buffalo. Our study population included a wild buffalo herd, which was captured in Northern KNP during the early 2000s and relocated to a 900‐hectare enclosure in the center of the park, near Satara camp (Figure S1). The buffalo sample size varied during our study (*N* = 60–70) due to births and deaths. On average, 25.60 ± 0.10% of the herd were calves, 22.95 ± 0.09% were juveniles and 51.45 ± 0.19% were adults. Buffalo were free to graze and breed in the enclosure, and during extreme droughts, they had access to supplemental grass hay. Water was available to buffalo at a natural pan and a manmade water point (Figure S1). This “nearly natural” enclosure included numerous other species typical of the ecosystem (e.g., giraffe, zebra, warthogs, and small predators) while excluding megaherbivores (e.g., rhinos, elephants) and large predators (e.g., lions, leopards).

### 2.2 Buffalo captures and sedation procedures

Data collection spanned six observation periods (OPs) that occurred from March 2014 to May 2015 (Table S1). We captured buffalo to collect biological data and download association data from proximity‐logging collars at the end of each OP. Captures occurred five times per year, at two to three‐month intervals. We performed three active captures, in which buffalo were darted from a helicopter, and four passive captures during the dry season in which researchers filled man‐made water troughs that attracted buffalo into a fenced area with a remote‐controlled gate closure (Figure S1). At each capture, we darted small groups of buffalo using chemical immobilization procedures described by Couch et al. (2017).

### 2.3 Data collection: biological data and samples

At captures, we visually sexed sedated buffalo and determined individual age by incisor wear and tooth emergence (Jolles, 2007). We assigned each buffalo a body condition score (BCS) on a scale of 1–5, determined by palpation (following Ezenwa et al., 2009). We evaluated average horn width, proposed as a proxy for overall animal quality (Ezenwa & Jolles, 2008), by measuring the widest point of each buffalo's horns. We also measured average boss size (i.e., the fused base of the horns) and average testicle circumference at the widest point for male juveniles and adults. We determined female lactation status (0/1) by manually milking all teats (Beechler, 2013), and we assessed pregnancy status (0/1) by rectal palpation of the uterus. African buffalo gestational periods last approximately 11 months (Sinclair, 1977).

Throughout the study period, we also collected ear tissue samples (2–4 cm), which we used to determine genetic relatedness among individuals (Tavalire et al., 2018; details in Data S1).

### 2.4 Data collection: behavioral association data

In February 2014, we fitted the majority of buffalo aged over 6 months with Sirtrack proximity‐logging collars (Sirtrack Tracking Solutions, Havelock North, New Zealand), which record the identity of collars in close proximity in addition to the date, time, and duration of each encounter. Percentage coverage across the herd is provided in Table S1 of Data S1. Calves <6 months in age were not collared for ethical considerations, as their growth rate exceeded collar re‐fitting schedules. We programmed collars with a UHF range coefficient of 20 and a separation time of 240 s, which in a laboratory setting initiated an association when collars were within 1.22 m ± 0.46 m (mean ± SD), and terminated the association when collars exceeded a distance of 1.70 m ± 0.67 m for more than 240 s. We deemed this a reasonable representation of transmission distances for pathogens spread via close contact (e.g., respiratory viruses and bacteria; Olsen et al., 2003; Wells, 1934). While proximity collars allowed for near‐complete data collection without observation bias, we note that close contacts were inferred from data on physical proximity (Farine, 2015; Farine & Whitehead, 2015) rather than directly‐observed interactions.

### 2.5 Estimating association indices and social networks

We analyzed association data for 69 buffalo (males = 22, females = 47), including 18 calves (<1.5 years). We created a matrix of pairwise association indices for each of the six observation periods (OPs). The number of buffalo varied across OPs due to births, deaths, and collar malfunctions. Overall, matrices included an average of 39.33 buffalo (±SD: 14.11) and ranged from 17 to 53 buffalo (Table S1).

When cleaning association data, we excluded capture days and a 2‐day buffer after each intervention, and we removed 1 s encounters shown to skew results (Drewe et al., 2012). To reduce asymmetries in association matrices, we excluded encounters logged after one individual in a pair had a full proximity logger memory. For each pair of individuals *i* and *j*, we calculated an association index (AI) for a given OP as follows:

$${\mathrm{AI}}_{\mathrm{ij}}=\frac{C_{\mathrm{ij}}}{C_{\mathrm{ij}}+N_{\mathrm{ij}}}$$

in which *C*_*ij*_ refers to the summed duration (h) of association logged between individuals *i* and *j* during the observation period, and *N*_*ij*_ refers to the total hours during the observation period in which *i* and *j* were not in contact. Thus *T*_*ij*_, in which *T*_*ij*_ = $C_{\mathrm{ij}}$+$\;N_{\mathrm{ij}}$, refers to the total hours during the observation period in which individuals *i* and *j* could associate with each other (i.e., both had collars with available memory to record data). Because data were collected continuously by proximity collars with little to no observation bias or missing groups within the herd (Davis et al., 2018), we used the simple ratio index of proportion of time a dyad spent in close proximity (Farine & Whitehead, 2015) with the resultant index being a value between 0 and 1 that indicates the proportion of time a dyad spent in close proximity. These indices were used to create adjacency matrices.

When recording association data, ideally both collars in a pair would record identical information; however, previous studies demonstrate that collars vary in their abilities to transfer information (Boyland et al., 2013; Drewe et al., 2012). We reduced inter‐collar variation biases, specifically conflation of individual ID and error/strength of proximity collar, using a method similar to that proposed by Boyland et al. (2013). In brief, we assessed variation in reciprocal AIs in a given matrix to evaluate each collar's relative performance and to develop a measure of collar bias. We then corrected matrix AIs by scaling each collar's data according to its average bias across collars, resulting in a nearly identical pre‐ and post‐correction average AI for the matrix. Further details about collar corrections are provided in Data S1. Finally, we used matrices to develop association networks corresponding to the six OPs. In each network, nodes represented buffalo with available data for a given OP, and network edges were weighted according to the AI calculated for each dyad.

### 2.6 Statistical analysis: pairwise association models

To examine the effect of biological traits on association patterns, we fit pairwise AI data to a Bayesian logistic mixed‐effect model using a multimembership Markov chain Monte Carlo (MCMC) framework with the *MCMCglmm* package in R (Hadfield, 2010; Hart et al., 2022; R Core Development Team, 2010). This multimembership modeling framework included a node dependence term and accounted for the undirected nature of association measures (Hart et al., 2022). We examined the relationship between pairwise AIs (represented in the model as a proportion of time spent together for a given observation period) and the following pairwise predictor variables: age/sex (pairwise combinations of: adult female, adult male, juvenile, and calf), number of pregnant buffalo in pair (0, 1, 2), number of lactating buffalo in pair (0, 1, 2), difference in BCS, and genetic relatedness. Histograms of continuous data for pairwise difference in BCS and genetic relatedness each showed three peaks, prompting the conversion of these data into categorical variables.

For all statistical analyses: buffalo were grouped as calves (<1.5 years), juveniles (≥1.5 and ≤4 years), or adults (>4 years); BCS was averaged across measurements collected at the capture before and after each OP; a female's reproductive status was determined at the capture prior to a given OP. Categories describing pairwise difference in BCS included: low (<0.5), medium (0.5–1), and high (>1). Similarly, categories described genetic relatedness as low (<0.12: cousins and unrelated pairs), medium (0.12–0.36: half‐siblings and aunt‐niece level relationships), or high (>0.36: full siblings and parent‐offspring pairs; additional details in Data S1).

Horn width increases as an animal grows; therefore, for the pairwise association models we performed a linear regression of log(average age) on average horn width for buffalo captured at least three times, with different slopes for males and females. We used the resulting regression residuals, proposed as an indicator of individual quality (Ezenwa & Jolles, 2008), in subsequent analyses. We used a similar approach to calculate residuals for average testicular size and average boss size for male juveniles and adults captured at least three times. We expected that males with larger testicles and boss sizes may have increased mating access and contact rates.

We examined model fit for the pairwise association model with the following random effects: buffalo identity, pair identity, and observation period.

### 2.7 Statistical analysis: network centrality models

To evaluate the effect of host traits on individual connectivity, we calculated centrality metrics for buffalo in each network using the *igraph* and *sna* packages in R (Csardi & Nepusz, 2006; McFarland et al., 2010). Specifically, we calculated the following weighted metrics: *strength* (summed edge weights connected to a given node), *flowbetweenness* (proportion of times a node lies along the shortest path between pairs in the network), and *eigenvector* (a function of the connectedness of a nodes' associates; Freeman et al., 1991; Newman, 2010). While strength considers a node's immediate neighbors, flowbetweenness and eigenvector centrality also account for indirect connections. We chose these metrics based on studies that have shown that individuals with high flow‐betweenness, strength, and/or eigenvector centralities are more likely to contract and transmit pathogens (Corner et al., 2003; Gómez et al., 2013; Salathé et al., 2010). To assess variation in diversity of associates, we also calculated a filtered degree centrality metric. Degree typically sums the number of edges for a given node. However, because all six networks were fully connected (i.e., all buffalo associated with each other), we filtered out edges below each network's median association, effectively removing the weakest 50% of edges. Without a filter, all buffalo in the fully connected networks would have the same degree centrality for a given observation period. We summed the remaining edges for a given node to calculate *filtered degree centrality* (hereafter referred to as *degree*). Notably, *degree* is the only metric with a filtered approach. Previous epidemiological studies have shown that strength outperforms other centrality metrics (e.g., degree, betweenness, and eigenvector centrality) when predicting an individual's infection risk (Christley et al., 2005) and predicting outbreak size based on an index case's centrality (Salathé et al., 2010). Additionally, multiple testing of several correlated metrics can lead to an increased false discovery rate (type I error; Benjamini & Hochberg, 1995). Given that *strength* was strongly correlated with *eigenvector* (*r*_s_ = .86, *n* = 241, *p* < .001) and *flowbetweenness* (*r*_s_ = .79, *n* = 241, *p* < .001), but only moderately correlated with *degree* (*r*_s_ = .54, *n* = 241, *p* < .001), our models focused on testing how life history traits affect individual *strength* and *degree*.

We fit individual centrality data to node‐level permutation‐based regressions in R (30,000 permutations/test; following Rushmore et al., 2013). Using a separate model for each centrality metric (i.e., *strength* and *degree*), we first examined a set of global models that included all buffalo across all observation periods and tested significant relationships between individual centrality and the following biological traits: sex (M/F), age (continuous), average BCS (continuous), lactation status (1/0), pregnancy status (1/0), and an age:sex interaction. Horn width data were only available for adults and juveniles; thus, we established a second set of models that only included data for adult and juvenile buffalo and examined relationships between individual centrality and sex, age, average BCS, horn width residuals (continuous), and age:sex. Lastly, testicular size and boss size were only available for adult and juvenile males; thus, we used a final model set to assess relationships between individual centrality and age, average BCS, testes size residuals (continuous), and boss size residuals (continuous). Each model incorporated a categorical parameter for the observation period to control for repeated measures and temporal effects. We applied a Bonferroni correction to model outputs to account for multiple testing of two centrality metrics and considered relationships of *p* < .025 (i.e., *p* < .05/2) to be significant.

### 2.8 Examining variation in individual R_0_ and pathogen invasibility of the herd

We examined how the observed connectivity patterns might affect a pathogen's invasibility—ability to invade and spread within the herd—for a range of pathogen infectious periods and transmission efficiencies. Here we make a simplifying assumption that there is a minimum time in close contact needed to achieve transmission. The mean and variance of *individual R*_0_ values (i.e., where *individual R*_0_ refers to the number of expected secondary infections arising from a given individual) define a pathogen's ability to invade and spread within a population (Lloyd‐Smith et al., 2005). Thus, we used an iterative approach to calculate *individual R*_0_ values for each buffalo across a range of infectious periods (range: 1–7 days) and transmission efficiencies (defined as the minimum amount of time a dyad needs to spend in close contact for pathogen transmission to occur; range: 30–600 min). Specifically, for a given buffalo and observation period (OP) we randomly selected a timeframe of association data corresponding to a given infectious period (e.g., 2 days). Then for a given transmission efficiency, we determined the buffalo's *individual R*_0_ as its number of transmission‐relevant contacts (i.e., the number of contacts exceeding the minimum duration for transmission). We iterated this process 50 times per parameter combination (buffalo × OP × infectious period × transmission efficiency), and averaged outcomes across iterations and OPs to determine a *mean individual R*_0_ (hereafter referred to as *v*) for each buffalo at each pathogen infectious period and transmission efficiency. Our selection of infectious periods and transmission efficiencies were based on available estimates of disease parameters relevant to our study system (details in Data S1).

In populations with homogenous contact patterns, pathogens tend to invade if the mean *v* > 1 (i.e., *R*_0_ > 1; Anderson & May, 1991); however, invasion likelihood is highly dependent on variation in individual infectiousness around a population's mean *v* (i.e., *R*_0_), and this variation can be heavily influenced by contact heterogeneity (Lloyd‐Smith et al., 2005). Thus, for each pathogen infectious period and transmission efficiency, we calculated the proportion of the herd with *v* > 1 to visualize how the observed association patterns affect pathogen invasibility at the herd level.

## 3 RESULTS

### 3.1 Close‐contact heterogeneity

**FIGURE 1:**
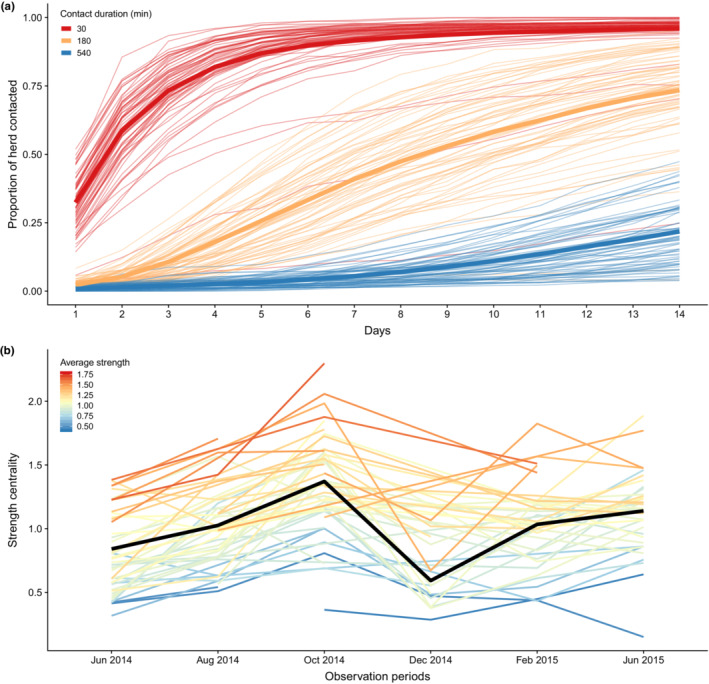
Buffalo showed considerable heterogeneity in close‐contact patterns, and individual buffalo centrality was consistent over time. (a) The average cumulative proportion of the herd each buffalo “contacted” within 1 m (i.e., individual degree/total collared buffalo) over randomly‐selected 14‐day periods is shown for three minimum contact durations (red: 30 min, orange: 180 min, blue: 540 min). Each thin line represents a single buffalo, with a thick line showing the herd average for each minimum contact duration. (b) Each colored line indicates the strength centrality for a single buffalo at each of the observation periods (OPs) the buffalo was observed. Lines are colored according to each buffalo's average (mean) strength centrality across OPs (red: highest average strength, blue: lowest average strength). The black line shows the mean herd strength centrality at each OP. Corresponding modularity (degree of subdivision) showed relatively less variation during the study period (see Figure S3). Apparent decreases in strength centrality in Observation Period 4 (December 2014) are likely due to unrelated collar failures which resulted in only 17 individuals being sampled during this observation period (Table S1).

**FIGURE 2:**
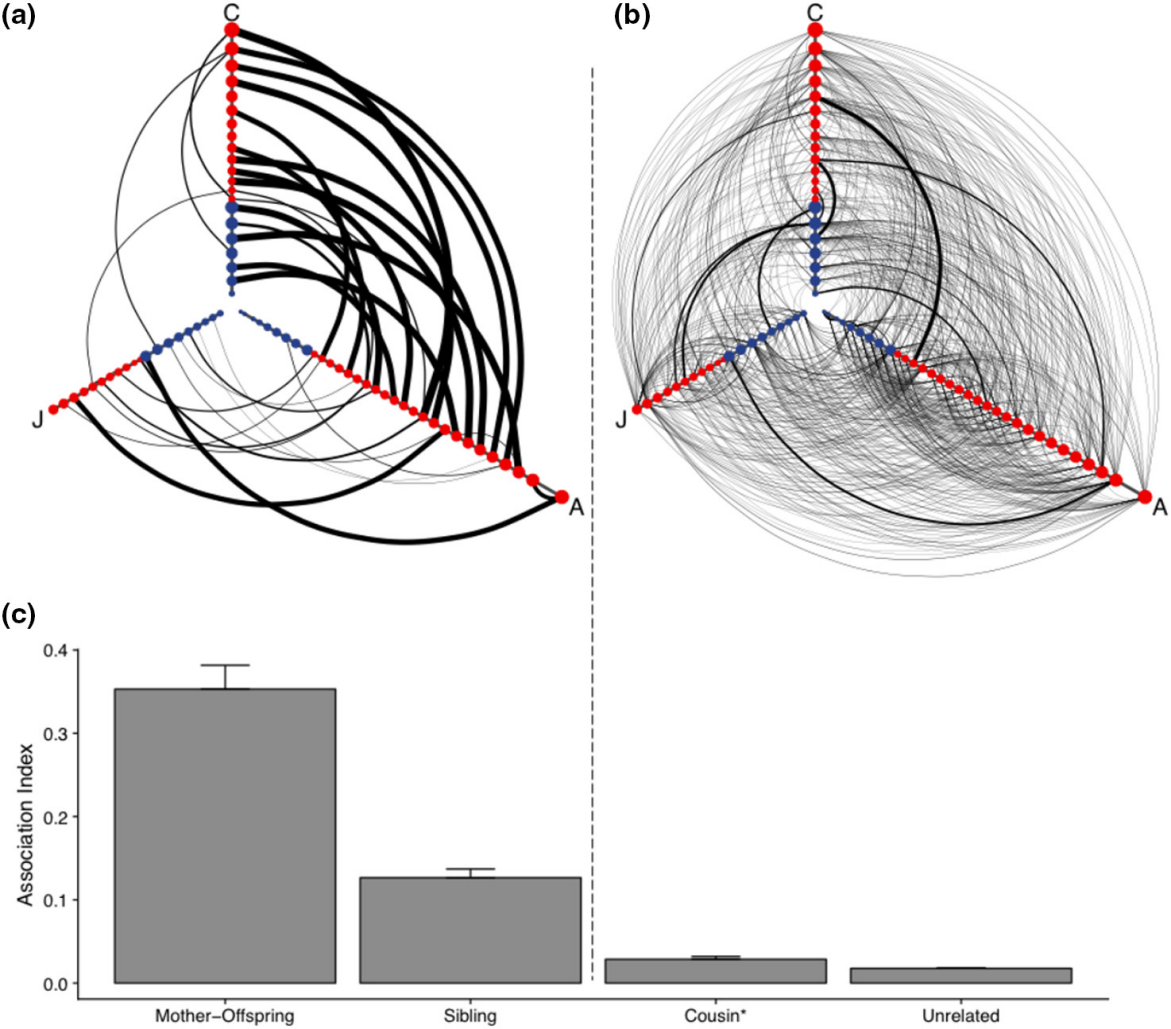
Relatedness, age, and sex predict connectedness among buffalo. Hive plots show connections between (a) highly related buffalo pairs (i.e., mom‐offspring and full‐sibling) and (b) all other buffalo pairs. Buffalo are represented as circular nodes along three age‐based axes (A, adult; C, calf; J, juvenile). Node colors indicate sex (red: female, blue: male), with larger nodes having a higher strength (averaged across observation periods). Weak connections below the median association index (across all pairs) are not visualized. Individuals with data spanning two age classes (e.g., juveniles who become adults during the study period) are represented as two nodes, one for each age class with node size and edge weights corresponding to associations observed for the individual at each age status. (c) Bar plots show mean association indices (+SE) across relatedness categories (after averaging association indices for each pair across observation periods). Categories shown include: mother‐offspring pairs (*N* = 30), full sibling pairs (*N* = 4), cousin pairs (*N* = 58), and unrelated pairs (*N* = 1438). *Pairs in the category “cousin” include cousins and half‐siblings (sharing one parent).

After removing capture and buffer periods, our dataset included an average of 5046 h (roughly 210 days; SD: 2077 h) of proximity collar data per buffalo during a 15‐month study period. The buffalo herd was well‐connected, with a single component (entirely connected network) for each two‐month observation period (OP). In fact, most buffalo associated with >75% of herd‐mates for at least 30 min within a 5‐day period (averaged across daily intervals selected randomly from each OP; Figure 1a). However, buffalo showed striking heterogeneity in their level of connectivity (Figures 1 and 2), including the rate with which they acquired connections within the herd (Figure 1a). Across OPs, randomly selected dyads spent an average of 2.74% of their time associating within a ~1 m distance, with considerable diversity across buffalo pairs (range: <0.01%–82.02%). Buffalo demonstrated general temporal consistency in network centrality such that buffalo with high or low centrality at the beginning of the study period tended to maintain their relative level of connectedness throughout the study duration (Figure 1b).

### 3.2 Drivers of close‐contact heterogeneity

**FIGURE 3:**
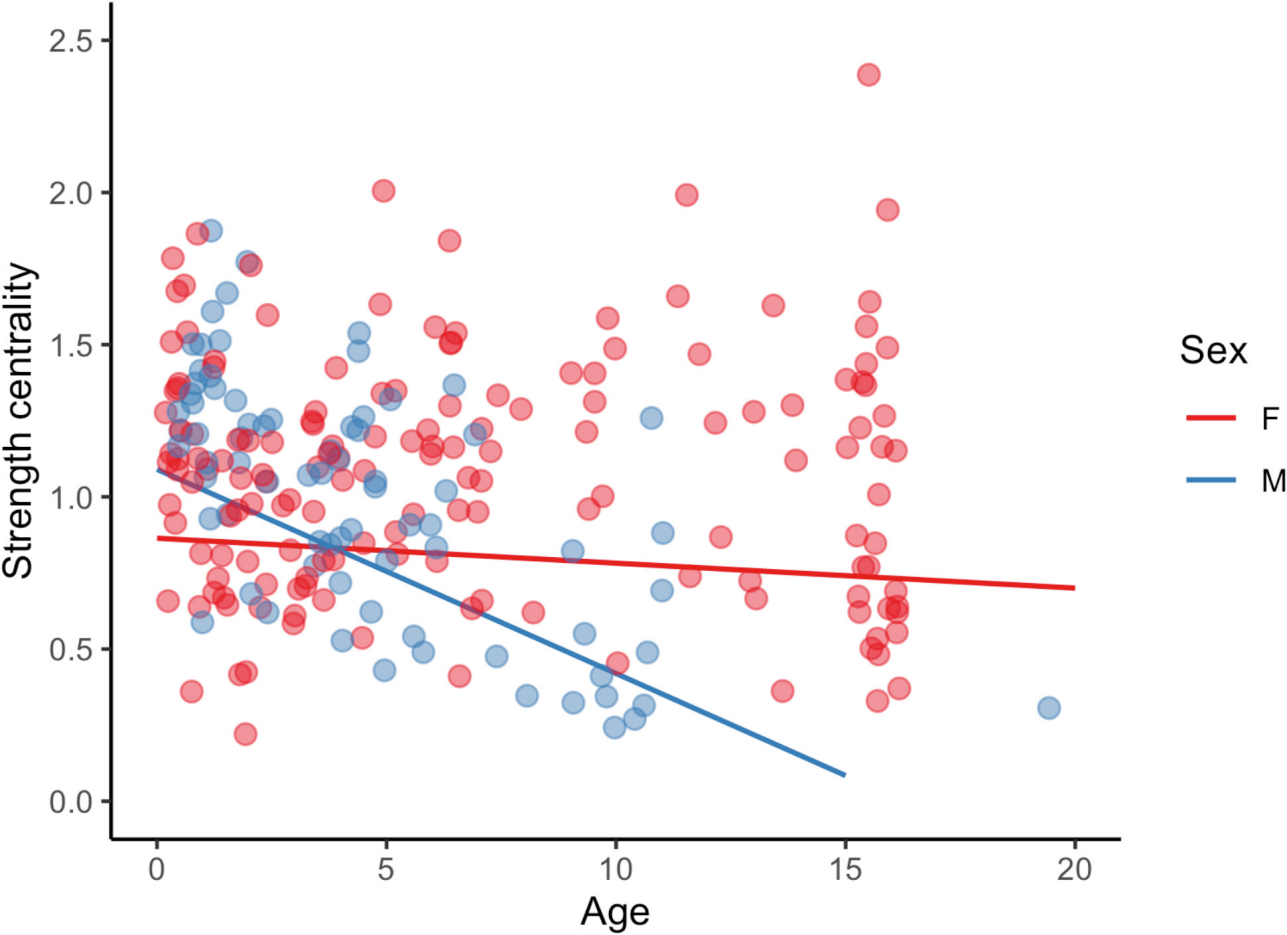
Age and sex significantly affected buffalo centrality. Scatterplots visualize the age‐sex interaction for strength centrality. Points show centrality data for females (red) and males (blue), and model predictions are shown as lines for females (red) and males (blue).

Network‐based models revealed that key buffalo traits were significantly associated with connectivity. Overall, the best predictors of connectedness were pairwise relatedness, individual age, individual sex, and female reproductive status (Figures 2 and 3). Relatedness played the biggest role in determining the likelihood of buffalo interacting (Table 1, Figure 2). Dyads with a high level of relatedness (i.e., full siblings, parent‐offspring pairs) or medium level of relatedness (i.e., half‐siblings, aunt‐niece pairs) were significantly more likely to associate than dyads with a low level of relatedness (i.e., cousin pairs, unrelated pairs; Table 1, Figure 2). In fact, highly related buffalo were over 14 times more likely to associate than those with a low level of relatedness (Table 1). Mother‐offspring pairs associated substantially more than other pairs (Figure 2), with mother‐calf pairs being within ~1 m of each other 53.7% of the time on average, whereas unrelated pairs only associated an average of 1.7% of the time. Despite a small number of full sibling pairs in our dataset, we found that calves and juveniles associated more with full siblings (mean: 13.8% of the time, *N* = 4 pairs) than with cousins/half‐siblings (mean: 2.8%, *N* = 37 pairs) or unrelated buffalo (mean: 1.7%, *N* = 255 pairs; Figure 2, Figure S2). Observed father‐offspring pairs associated less than 1% of the time; however, this calculation is based on a small sample size of 14 offspring who all shared the same father; no other fathers existed in the study herd.

**TABLE 1:** Effect of biological host traits on pairwise buffalo associations.

Factor	Posterior mean	CI	*p*‐value	OR
Intercept	−4.280	(−4.78, −3.70)	<.001	
**Group (AM: C)**	−0.198	(−0.37, −0.03)	**.028**	0.82
**Group (AM: J)**	−0.220	(−0.36, −0.09)	**<.001**	0.8
Group (AM: AM)	0.073	(−0.18, 0.31)	.561	1.08
**Group (AF: C)**	0.295	(0.00, 0.57)	**.042**	1.34
Group (AF: J)	0.007	(−0.24, 0.28)	.977	1.01
Group (AF: AM)	0.012	(−0.27, 0.30)	.953	1.01
Group (AF: AF)	0.279	(−0.20, 0.79)	.266	1.32
Group (J: C)	−0.009	(−0.13, 0.11)	.876	0.99
**Group (C: C)**	0.261	(0.06, 0.49)	**.018**	1.3
Lactation: 1	0.021	(−0.04, 0.08)	.501	1.02
Lactation: 2	0.131	(−0.02, 0.25)	.066	1.14
**Pregnant: 1**	0.122	(0.06, 0.19)	**<.001**	1.13
**Pregnant: 2**	0.170	(0.03, 0.32)	**.022**	1.19
**Relatedness: med**	0.190	(0.14, 0.25)	**<.001**	1.21
**Relatedness: high**	2.668	(2.56, 2.78)	**<.001**	14.41
Diff in BCS: med	0.019	(−0.02, 0.06)	.346	1.02
Diff in BCS: high	0.016	(−0.08, 0.12)	.741	1.02

*Note*: The posterior mean, 95% credible interval, *p*‐value based on MCMC sampling, and odds ratios (OR) are shown for fixed effects. Random effects include buffalo identity and observation period; pair identity was not included in the final model due to improved model fit after removal. J, J is the baseline age‐sex category. Bolded values indicate significant relationships *P* < .05.

Abbreviations: AF, adult female; AM, adult male; BCS, body condition score; C, Calf; J, Juvenile.

After relatedness, buffalo age and sex had the greatest impact on herd connectivity. Our network centrality model showed that an age‐sex interaction significantly affected buffalo strength and degree centrality (Table 2). Connectivity decreased with age, but this effect was much stronger for males than for females, who maintained a relatively consistent centrality over their lifetimes (Table 2, Figure 3). Males started to experience a lower centrality than females around age 4–5 years (Figure 3). In general, calves had the highest centrality, and dyads with a calf had among the highest association rates. For example, AF‐C and C‐C pairs were each approximately 30% more likely to associate than J‐J pairs (baseline; Table 1; see Table 1 caption for abbreviations). Dyads that included an adult male and a young buffalo (i.e., AM‐C, AM‐J pairs) tended to have lower association indices than corresponding pairs without an adult male (e.g., AF‐C, C‐C, J‐J pairs; Table 1).

**TABLE 2:** Effect of host traits on individual centrality during six observation periods (*N* = 235).

Factor	Strength centrality		Degree centrality	
	*β*	*p*‐value	*β*	*p*‐value
Intercept	1.01	.438	30.54	.095
Sex (M)	0.23	**.006**	6.33	**.005**
Age	−0.01	.149	−0.13	.273
Average BCS	−0.06	.230	−2.21	.140
Pregnant (1)	0.11	.113	4.32	*.036*
Lactating (1)	0.15	*.041*	2.15	.179
Sex (M): Age	−0.06	**<.001**	−1.08	**.004**

*Note*: Italicized values indicate significant relationships (*p* < .050). Bolded values indicate significant relationships after Bonferroni correction (*p* < .025). See Data S1 for observation period estimates and *p*‐values (Table S2).

Abbreviations: BCS, body condition score; M, male; 1, “yes” for 1/0 binomial indicators.

Female reproductive status had a marginal impact on buffalo connectivity. Most notably, pairs with at least one pregnant female were significantly more likely to associate than pairs without a pregnant female (Table 1). Pregnant females also tended to have a higher degree centrality, although this test was not significant after a Bonferroni correction (*p =* .036, Table 2). While lactation status did not significantly affect the likelihood of buffalo associating (Table 1), lactating buffalo tended to have higher strength centrality (not significant after a Bonferroni correction, *p* = .041; Table 2). We did not observe an effect of testicular size, BCS, horn width, or boss size on connectivity (Tables S3 and S4).

### 3.3 Individual *R*_0_ (*v*) and pathogen invasibility of the herd

**FIGURE 4:**
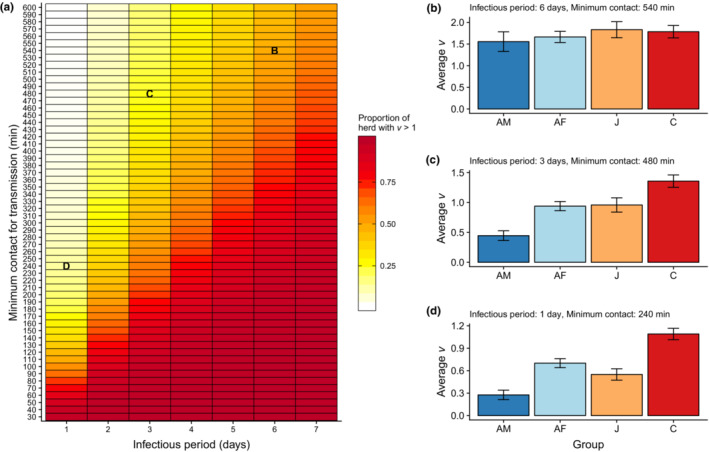
Herd association patterns shape the pathogen invasibility landscape and life history traits drive variation in mean individual R_0_ (*v*) for pathogens with relatively short infectious periods. (a) The proportion of the herd with *v* > 1 is shown for a range of pathogen infectious periods and transmissibilities (i.e., minimum minutes of association required for pathogen transmission). Bar plots, broken down by age and sex (AF, adult female; AM, adult male; C, calf; J, juvenile), show average *v* (mean ± SE) for pathogens with infectious periods and transmissibilities that are (b) long/low, (c) medium, and (d) short/high.

Our calculations of *v* and the proportion of the herd with *v* > 1 indicate that close‐contact heterogeneities drive invasion likelihoods in our study herd for pathogens with a range of infectious periods and transmission efficiencies (Figure 4a). In particular, upwards of 75% of the herd had *v* > 1 (indicated by dark red: Figure 4a) when minimal to moderate contact was required for transmission, depending on the infectious period. Pathogens with short infectious periods (<2 days) and very long contact requirements for transmission resulted in a few individuals having *v* > 1 (indicated by white/pale yellow: Figure 4a). The remaining portions of Figure 4a (indicated by yellow/light orange) show pathogen infectious periods and contact durations for which ~25%–75% of the herd had *v* > 1. Further investigation into a subset of parameter combinations revealed that age and sex were often predictive of *v*, such that on average, calves had *v* > 1 and adult males had *v* < 1: Figure 4c,d when there was considerable heterogeneity in associations (e.g., yellow regions: Figure 4a); however, at parameter combinations with more homogenous connectivity patterns, age, and sex had little effect on *v*, and all age‐sex combinations had a mean *v >* 1 (Figure 4b).

## 4 DISCUSSION

Our study strengthens challenges to the assumption within epidemiological models that gregarious ungulate herds are well‐mixed with respect to pathogen transmission. While our network analyses of African buffalo indeed revealed a well‐connected herd, we found considerable heterogeneity in the duration of close‐contact interactions among buffalo. Herd connectivity was highly dependent on interval length: at short time scales, only a small subset of individuals contacted one another, whereas at longer time scales, connectivity saturated to panmixia. Individual centrality was stable across observation periods and key buffalo traits predicted connectivity, indicating that different classes of individuals likely play contrasting roles in contact‐driven processes, such as pathogen transmission. For example, pathogens with short infectious periods require highly connected individuals for invasion to occur. Our study herd is regularly exposed to a range of respiratory pathogens that range in dynamic behavior from endemic to cyclical to sporadic (Glidden et al., 2021). By visualizing the proportion of the herd with *v* > 1 for a range of pathogen infectious periods and transmission efficiencies, we found that the level of contact heterogeneity we observed in buffalo would be sufficient to shape the likelihood of pathogen invasion, even within our well‐connected and highly gregarious study herd.

We identified several life history traits that significantly affected individual connectivity, including pairwise relatedness, individual age and sex, and female reproductive status. Genetic relatedness was the strongest predictor of buffalo association rates, with mother‐offspring and full‐sibling dyads being significantly more likely to associate than other buffalo pairs. Kinship and kin selection are important drivers of behavioral associations in the animal kingdom, which is particularly evident among primates (Maestripieri, 2018; Silk, 2002; Städele et al., 2016) and birds (Krakauer, 2005; Leedale et al., 2020). Less is known about how kinship affects social patterns of large‐bodied herd‐living animals. Some species show aggregation among close relatives (e.g., elephants: Chiyo et al., 2011; Wittemyer et al., 2005), whereas others do not (e.g., elk: Vander Wal et al., 2012). For species that associate closely with kin, relatedness might predict pairwise infection likelihoods, as demonstrated for bovine tuberculosis infections among closely related white‐tailed deer (Blanchong et al., 2007) and African swine fever in wild boar (Podgórski et al., 2022).

Age and sex also significantly affected buffalo connectivity. Buffalo became less centrally connected with age, with a steeper decline for males than for females. Dyads including one adult male had among the lowest association rates. These findings support previous observations that adult male buffalo become less social around puberty (4–5 years) as they leave the breeding herd to join bachelor herds (Prins, 1996; Sinclair, 1977; Turner et al., 2005). Contrastingly, calves held relatively central positions in the social network, and dyads including at least one calf had among the highest association rates (other than AM‐C pairs). It is possible that early exposure to diverse social interactions may increase social competence and offer fitness benefits later in life, as has been suggested for a range of wildlife species (McDonald, 2007; Stanton & Mann, 2012; Thompson, 2019; Vander Wal et al., 2015). However, diverse social exposures may come at a cost if they simultaneously increase pathogen exposures.

Reproductive status played a small role in connectivity, such that pairs with one or two pregnant females were significantly more likely to associate than pairs without a pregnant female. While not significant after Bonferroni correction, pregnant and lactating buffalo also tended to be more central in the social networks. Female reproductive status has been linked to association patterns in a handful of wild and domestic ungulates. For example, Swain et al. (2015) found that pregnant and maternal (post‐calving) beef cows preferentially associated with individuals of the same status, with an immediate switch in preferred associates after calving. Networks of Grevy's zebra showed that females assorted according to their lactation status (Sundaresan et al., 2007), with similar findings in dairy cows (Boyland et al., 2016). Water and energetic needs typically vary with the reproductive stage and could drive association preferences (Boyland et al., 2016; Sundaresan et al., 2007). Relatively central positions in the social network may increase pathogen exposures for mothers and calves; however, these costs might be offset if assortative mixing allows mothers more time to graze or strengthens bonds that protect the young. Lastly, we did not observe an effect of testicular size, horn width, or boss size on buffalo connectivity, after controlling for age.

The observed herd connectivity patterns define the invasibility landscape for pathogens that vary in their infectious periods and transmission efficiencies. Specifically, our estimates of *v* indicated that index case centrality and herd connectivity would drive the invasion potential for pathogens with short infectious periods (1–4 days) and moderate close‐contact durations required for transmission. Contrastingly, the within‐herd structure would have minimal impact on invasion likelihood for pathogens with long infectious periods and minimal association requirements, as the majority of the herd had a *v* > 1. Similarly, briefly, infectious pathogens with lengthy contact requirements resulted in a majority of buffalo being unsuitable as an index case (with *v* < 1). Buffalo and cattle diseases range widely in their transmission efficiencies, but little is known regarding how much time in close contact is required for transmission to occur. Charleston et al.'s (2011) study of direct FMDV transmission events determined that positive cattle at peak shedding can infect susceptible cattle within 2 h. They subsequently exposed naïve cattle to FMDV‐positive cattle in 8‐h time periods, which resulted in roughly a quarter of susceptible cattle becoming infected. Thus, while the average duration of transmission‐relevant contacts is not known, it appears that FMDV can transmit in brief time periods (<2 h), but it could take upwards of 10 h of close contact for transmission to occur. While little is known about the amount of contact required for transmission of most pathogens in ungulates, our results suggest herd connectivity could affect the invasion likelihood for FMDV and several respiratory viruses that have relatively short infectious periods with viral shedding often peaking in the first couple days after infection (see supplemental text: Charleston et al., 2011; Grissett et al., 2015). For pathogens with short infectious periods and moderate close‐contact durations, our analyses revealed that age and sex of the index case affected invasion likelihood, with calves being the most likely to have *v* > 1 and adult males being the least likely to have *v* > 1. This finding highlights the important role that susceptible calves may play in pathogen transmission within the herd. Herd structure and life‐history traits of the index case should matter less for the invasion potential of pathogens with very long infectious periods, like *Mycobacterium bovis*, which provide ample time for the network to fully saturate. We note that our emphasis here was on behavioral drivers of herd invasibility by pathogens. We have not addressed how the contact network structure we observed might influence other disease dynamic outcomes, such as the time to epidemic peak, outbreak size, or persistence time of relevant pathogens. Future work could address these questions through the development of an agent‐based model or network‐based model using a SIR framework.

While our study focused on behavioral drivers of *v*, it is important to point out that physiological heterogeneities can also affect *v* (Manlove et al., 2017). Specifically, individual differences in immunity can affect pathogen shedding and recovery rates, ultimately impacting an individual's level and duration of infectiousness (VanderWaal & Ezenwa, 2016). While behavior does not explain the whole picture, studies comparing contact networks and individual infection status show that connectivity can often serve as a reasonable proxy for estimating transmission potential across a range of host‐pathogen systems (Corner et al., 2003; Godfrey et al., 2009; Raulo et al., 2021; Tung et al., 2015). For example, a study of wild reticulated giraffe found that a giraffe's position in the social network predicted its position in an *Escherichia coli* transmission network, with social hubs also acting as transmission hubs (VanderWaal, Atwill, et al., 2014). Additionally, epidemiological models have shown that outbreaks tend to be more widespread if the index case is well connected whereas a pathogen may fail to invade if the index case is more peripheral (Lloyd‐Smith et al., 2005; Rushmore et al., 2014). In our study, buffalo calves emerged as the most widely connected animals in the herd; due to limited prior exposures, they are also among the most susceptible animals (particularly after maternal immunity has waned), underscoring the central role calves are likely to play in pathogen transmission within buffalo populations. Indeed, the propensity for gregarious and highly susceptible young‐of‐the‐year to spread infections in herd‐living ungulates may present an efficient mechanism for density‐dependent population regulation, as high recruitment may catalyze increased exposure of the herd to infectious pathogens. To better understand the synergistic effects of host sociality and physiology on pathogen invasion and transmission dynamics, our upcoming work will explore relationships between connectivity, immunity, and infection status for respiratory diseases among buffalo in this herd.

A notable limitation of our study is its focus on directly‐transmissible pathogens. Several studies have shown that environmental transmission (indirect transmission) can also play a key role in some systems (e.g., Beerens et al., 2021; Breban et al., 2009). In these systems, invasibility may be more dependent upon pathogen viability in the environment and host habitat use than host contact rates. Future work could quantify the relative effect of contact heterogeneity versus space use on the invasibility of pathogens that use multiple routes of transmission. As an additional limitation, while our study herd was in a large “nearly natural” enclosure with other species typical of the ecosystem, there remain differences with free‐ranging buffalo populations. In particular, predator exclusion, occasional supplemental feeding, and access to only a single water source in the dry season could affect social dynamics in our study herd.

In conclusion, we found considerable close‐contact heterogeneity in an African buffalo herd, with particular life‐history traits predicting association patterns. These findings challenge the assumption within epidemiological models that ungulate herds are well‐mixed with respect to pathogen transmission. Our study further shows that heterogeneity among individuals drives invasion likelihood at time scales relevant to infectious pathogens, even within a highly‐connected and gregarious population. For a range of pathogen parameters, well‐connected age‐sex classes were more likely to have *v* > 1, making index cases with key characteristics more likely to spark outbreaks. Our data provide quantifiable differences among age‐sex classes that can be used to parameterize network‐based infectious disease models for evaluating pathogen transmission and control in this epidemiologically important wildlife host species. Finally, our study provides a framework that links host connectivity and infectious disease biology to characterize a study population's pathogen invasion landscape and identify classes of superspreading hosts.

## AUTHOR CONTRIBUTIONS

**Julie Rushmore:** Conceptualization (equal); data curation (equal); formal analysis (equal); investigation (equal); methodology (equal); visualization (equal); writing – original draft (equal); writing – review and editing (equal). **Brianna R. Beechler:** Conceptualization (supporting); data curation (supporting); formal analysis (supporting); methodology (supporting); project administration (supporting); resources (supporting); writing – review and editing (supporting). **Hannah Tavalire:** Data curation (supporting); writing – review and editing (supporting). **Erin E. Gorsich:** Formal analysis (supporting); writing – original draft (supporting). **Bryan Charleston:** Funding acquisition (equal); resources (supporting); writing – review and editing (supporting). **Anne Devan‐Song:** Writing – review and editing (supporting). **Caroline K. Glidden:** Writing – review and editing (supporting). **Anna E. Jolles:** Conceptualization (equal); data curation (equal); funding acquisition (equal); methodology (supporting); project administration (equal); resources (equal); writing – review and editing (supporting).

## CONFLICT OF INTEREST STATEMENT

The authors have no conflicts of interest to declare.

## Supporting information


Data S1:

## Data Availability

The data that support the findings of this study are openly available in Dryad at https://doi.org/10.5061/dryad.9zw3r22m0, DOI reference number https://doi.org/10.5061/dryad.9zw3r22m0.
