# Supplementary material for: The heterogeneous herd: Drivers of close‐contact variation in African buffalo and implications for pathogen invasion
Source: Ecol Evol. 2023 Aug 22;13(8):e10447. doi: 10.1002/ece3.10447 (PMC10445036; doi:10.1002/ece3.10447)
Supplement: Supplementary file 1 — Data S1: [file ECE3-13-e10447-s001.docx]

**Supporting Information**

**The heterogeneous herd: drivers of close-contact variation in African buffalo and implications for pathogen invasion**

Julie Rushmore, Brianna R. Beechler, Hannah Tavalire, Erin Gorsich, Bryan Charleston, Anne Devan-Song, Caroline K. Glidden, Anna E. Jolles

**I. Supporting Text**

***Determining genetic relatedness***

We extracted 100-200ng/ml genomic DNA from dried ear tissue samples (DNeasy blood & tissue kit, Qiagen) and prepared individual libraries for sequencing using type IIB restriction associated DNA (2bRAD) methods, detailed in Wang *et al.* (2012). Briefly, this method uses a type IIB restriction endonuclease (Alf1; Thermo Scientific #ER1801) to extract thousands of 36bp reads from across the genome. After quality filtering, we used SHRiMP (Rumble *et al.* 2009) to map each individual to the *de novo* assembly of AlfI sites, and we filtered the resulting matches for statistically weak or ambiguous alignments following parameters described by the software authors. We determined genotypes at each AlfI site with > 5x coverage, then filtered out monomorphic loci. We allowed for 10% missing data at any given locus and one polymorphism per tag. Animals that were genotyped at 33% or fewer (< 11800) loci were removed from the dataset. The analysis pipeline outlined above was developed by Eli Meyer (available at https://github.com/Eli-Meyer). Markers were discarded if they violated Hardy Weinberg Equilibrium (p < 0.0001) or had a minor allele frequency less than 5%. Ultimately, filtering yielded samples genotyped at 2505 SNPs.

***Assigning pairwise relatedness categories***

We determined marker-based pairwise relatedness (r) using the 2505 SNPs to bin dyads into relatedness classes (e.g., parent-offspring, full siblings, cousins, unrelated). Sibling and parent-offspring pairs were assigned in the program COLONY using default settings (Jones & Wang 2010). Cow-calf assignments showed 100% accuracy with assignments based on behavioral observations during capture. We then estimated identity by decent-based (IBD) r values for all pairwise relationships using the maximum likelihood estimator (Milligan 2003) in the R package ‘*related*’ (Pew *et al.* 2015). This allowed us to infer paternity and estimate relatedness among pairs that could not be inferred directly through pedigree reconstruction or observation (e.g., cousins if one parent was missing). Marker-based pairwise relatedness (r) has been shown to vary widely outside of expected pedigree-based average values due to recombination (Queller & Goodnight 1989; Lynch & Ritland 1999), thus we used marker-based r estimates for the subset of known cow-calf pairs identified by COLONY and lower-level relationships inferred through pedigree reconstruction to create cutoffs in the complete pairwise r distribution for relationship bin assignments. Using these cut-offs, we were able to assign previously cryptic pairwise relationships into categorical bins.

**Selection of infectious periods and transmission efficiencies**

While little is known about pathogen shedding rates in African buffalo, numerous experimental studies explore infectious periods for relevant cattle diseases. A review of respiratory disease challenge experiments in cattle determined that many respiratory viruses pertinent to buffalo (e.g., Bovine Herpesvirus Type 1, BHV-1; Bovine Viral Diarrheal Virus, BVDV; Parainfluenza-3, PI-3; Bovine Respiratory Syncytial Virus, BRSV) are shed in cattle for roughly 10 days, with peak shedding tending to occur early, around the 2^nd^-3^rd^ day of the infectious period (Grissett, White & Larson 2015). Notably, these estimates may be longer than the true values, as infectious periods inferred from viral isolation (e.g., in blood) can be longer than durations for which real transmission events are likely to occur (Charleston *et al.* 2011). An experimental study of direct FMDV transmission between cattle dyads kept in close contact (4 x 4.8 x 2.6m enclosures) estimated that FMDV has a mean infectious period of 1.7d (Charleston *et al.* 2011).

**Proximity collar corrections and matrices**

We reduced inter-collar variation biases using a method similar to that proposed by Boyland et al. ([2013](https://docs.google.com/document/d/1608zRNszDVcS5_XnKcu3cUUBUDb2kCEIl6uPqS6T4oc/edit#heading=h.1t3h5sf)). We assessed variation in reciprocal AIs in a given matrix to evaluate each collar’s relative performance/strength and to develop a measure of collar bias. We corrected matrix AIs by scaling each collar’s data according to its average bias across collars. We calculated association indices by dividing the amount of time two individuals spent together by the amount of time that pair had functioning collars. We then calculated the bias in sender/receiver contact durations across all pairs in the matrix and calculated the average bias generated by each collar. A correction factor (i.e., each collars’ average bias in a given matrix) was then applied across each collar for that association matrix. Spearman correlations were run before and after the correction to assess how the correction improves the sender/receiver contact agreement. We then averaged corrected sender and receiver durations to create a symmetric matrix.

**Sample R Code**

Below we provide R code for our final pairwise association model, which used the *MCMCglmm* package in R:

###### Set number of iterations and burnin #####

nitt=300000 # refers to number of iterations (referenced in model below)

burnin=10000 # refers to burn-in (referenced in model below)

###### Set priors #####

Priors<- list(R = list(V = 1, nu = 0.002), G = list(G1 = list(V =1, nu = 0.002), G2 = list(V =1, nu = 0.002))) # A list of priors (referenced in model below)

###### Final model #####

Model<- MCMCglmm(cbind(round(C_ij),round(N_ij)) ~ AgeSexGroup + LactationStatus + PregnancyStatus + RelatednessCategory + BCS_DifferenceCategory, random = ~idv(mult.memb(~Actor1+Actor2)) + ObservationPeriod, family="multinomial2", data=data, verbose = FALSE, prior=Priors, pr=TRUE, nitt=nitt, burnin=burnin)

**II. Supporting Tables**

**Table 1.** Details for each observation period including the start and end dates, sample size (N), and percent coverage.

| **Observation**  **period** | **Start date** | **End date** | **N^A^** | **Percent coverage^B^** |
| --- | --- | --- | --- | --- |
| 1 | Mar 9, 2014 | Jun 25, 2014 | 52 | 80 |
| 2 | Jul 4, 2014 | Aug 2, 2014 | 54 | 83 |
| 3 | Aug 16, 2014 | Oct 8, 2014 | 53 | 87 |
| 4 | Oct 31, 2014 | Nov 23, 2014 | 17 | 31 |
| 5 | Dec 14, 2014 | Feb 22, 2015 | 32 | 65 |
| 6 | Mar 1, 2015 | May 26, 2015 | 34 | 59 |

**^A^N** refers to the sample size of buffalo with adequate association data for inclusion in each social network

^B^**Percent coverage** indicates how much of the entire herd was included in each social network

**Table S2.** Effect of host traits on individual centrality during six observation periods. Coefficients (β) and *P*-values are shown for two global models (one for each centrality metric: degree, strength). For each model, N=235.

|  | **Strength Centrality** | |  | **Degree Centrality** | |  |
| --- | --- | --- | --- | --- | --- | --- |
| **Factor** | **β** | ***P*-value** |  | **β** | ***P*-value** |  |
| Intercept | 1.01 | 0.438 |  | 30.54 | 0.095 |  |
| Sex (M) | 0.23 | **0.006** |  | 6.33 | **0.005** |  |
| Age | -0.01 | 0.149 |  | -0.13 | 0.273 |  |
| Average BCS | -0.06 | 0.230 |  | -2.21 | 0.140 |  |
| Pregnant (1) | 0.11 | 0.113 |  | 4.32 | *0.036* |  |
| Lactating (1) | 0.15 | *0.041* |  | 2.15 | 0.179 |  |
| Sex (M): Age | -0.06 | **< 0.001** |  | -1.08 | **0.004** |  |
| Observation Period 2 | 0.17 | **0.024** |  | -0.99 | 0.334 |  |
| Observation Period 3 | 0.47 | **< 0.001** |  | -1.30 | 0.339 |  |
| Observation Period 4 | -0.27 | *0.045* |  | -19.64 | **< 0.001** |  |
| Observation Period 5 | 0.17 | 0.050 |  | -10.15 | **< 0.001** |  |
| Observation Period 6 | 0.33 | **< 0.001** |  | -5.23 | **0.015** |  |

M = Male; 1 = “yes” for 1/0 binomial indicators

Italicized values indicate significant relationships (*P* < 0.050)

Bolded values indicate significant relationships after Bonferroni correction (*P* < 0.025)

**Table S3.** Effect of host traits on adult and juvenile buffalo centrality during six observation periods. Coefficients (β) and *P*-values are shown for models (one for each centrality metric: degree, strength) that include only adult and juvenile buffalo. For each model, N=188.

|  | **Strength Centrality** | |  | **Degree Centrality** | |  |
| --- | --- | --- | --- | --- | --- | --- |
| **Factor** | **β** | ***P*-value** |  | **β** | ***P*-value** |  |
| Intercept | 1.07 | 0.404 |  | 32.99 | 0.063 |  |
| Sex (M) | 0.17 | 0.087 |  | 9.16 | **0.003** |  |
| Age | 0.01 | 0.165 |  | -0.07 | 0.359 |  |
| Average BCS | -0.10 | 0.102 |  | -2.67 | 0.110 |  |
| Horn width residuals | 0.00 | 0.363 |  | -0.01 | *0.455* |  |
| Sex (M): Age | -0.06 | **< 0.001** |  | -1.79 | **< 0.001** |  |
| Observation Period 2 | 0.19 | *0.025* |  | -0.46 | 0.436 |  |
| Observation Period 3 | 0.50 | **< 0.001** |  | -1.36 | 0.347 |  |
| Observation Period 4 | -0.31 | *0.033* |  | -18.34 | **< 0.001** |  |
| Observation Period 5 | 0.14 | 0.104 |  | -10.19 | **< 0.001** |  |
| Observation Period 6 | 0.39 | **< 0.001** |  | -4.16 | 0.056 |  |

M = Male; 1 = “yes” for 1/0 binomial indicators

Italicized values indicate significant relationships (*P* < 0.050)

Bolded values indicate significant relationships after Bonferroni correction (*P* < 0.025)

**Table S4.** Effect of host traits on adult and juvenile male buffalo centrality during six observation periods. Coefficients (β) and *P*-values are shown for two models (one for each centrality metric: degree, strength) that include only adult and juvenile male buffalo. For each model, N=53.

|  | **Strength Centrality** | |  | **Degree Centrality** | |  |
| --- | --- | --- | --- | --- | --- | --- |
| **Factor** | **Β** | ***P*-value** |  | **β** | ***P*-value** |  |
| Intercept | 1.10 | 0.339 |  | 33.73 | 0.227 |  |
| Age | -0.06 | **< 0.001** |  | -2.12 | **< 0.001** |  |
| Average BCS | -0.05 | 0.373 |  | 0.13 | 0.492 |  |
| Testicular size residuals | -0.03 | 0.051 |  | -0.93 | 0.063 |  |
| Boss size residuals | 0.01 | 0.385 |  | 0.27 | 0.326 |  |
| Observation Period 2 | 0.32 | 0.052 |  | 1.98 | 0.383 |  |
| Observation Period 3 | 0.56 | 0.011 |  | 5.41 | 0.253 |  |
| Observation Period 4 | -0.08 | 0.397 |  | -10.14 | 0.155 |  |
| Observation Period 5 | 0.22 | 0.154 |  | -6.50 | 0.173 |  |
| Observation Period 6 | 0.39 | 0.010 |  | -3.07 | 0.289 |  |

M = Male; 1 = “yes” for 1/0 binomial indicators

Bolded values indicate significant relationships after Bonferroni correction (*P* < 0.025)

**III. Supporting Figures**

**Figure S1.** The 900ha enclosure was roughly square shaped with two water points (indicated at yellow pointers), including a manmade water point and a natural pan (a). During passive captures animals were enclosed in the green capture bomas by a remote controlled gate after entering to drink water (b). The entire enclosure was double fenced to keep animals from coming into contact with other free-ranging wildlife in the park (c).

**
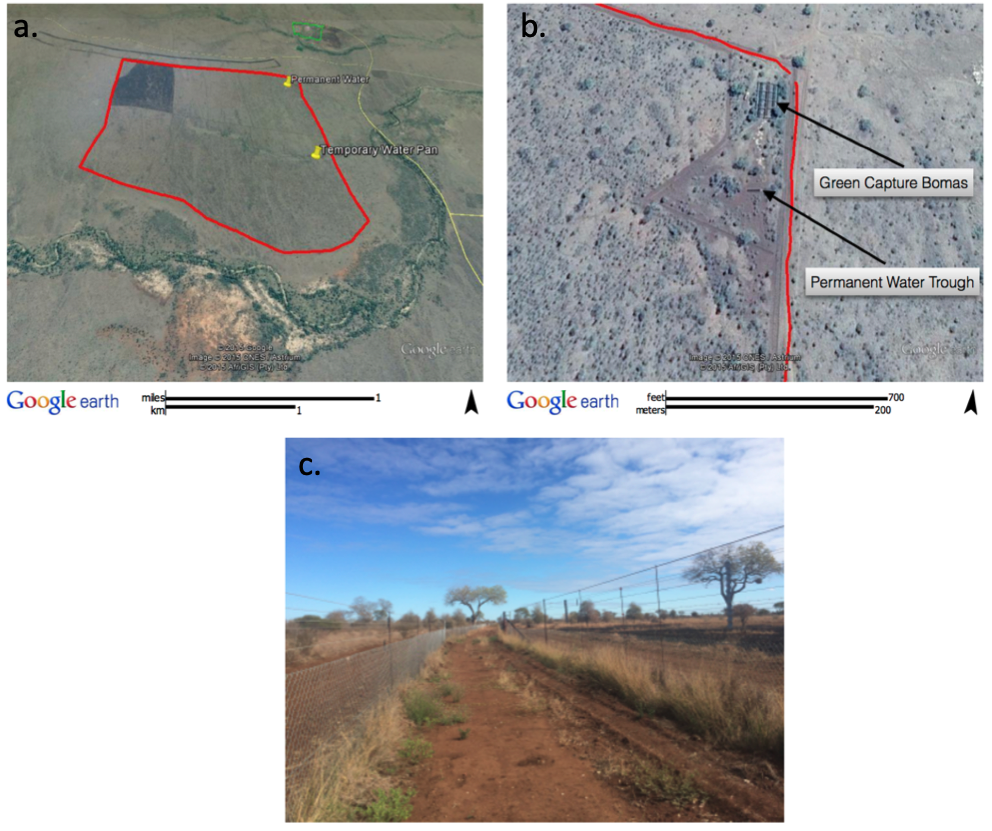
**

**Figure S2. Young buffalo associated more with full siblings than with other half-siblings/cousins or unrelated buffalo.** Bar plots show mean association indices (+ /- SE) across relatedness categories for young buffalo (calf-calf, calf-juvenile, and juvenile-juvenile pairs), after averaging association indices for each pair across observation periods. The sample sizes for each relationship category were as follows: full sibling (N = 4), cousin (N = 37 pairs), and unrelated (N = 255 pairs). *Pairs shown in the category “cousin” include cousins and half-siblings (sharing one parent).

**
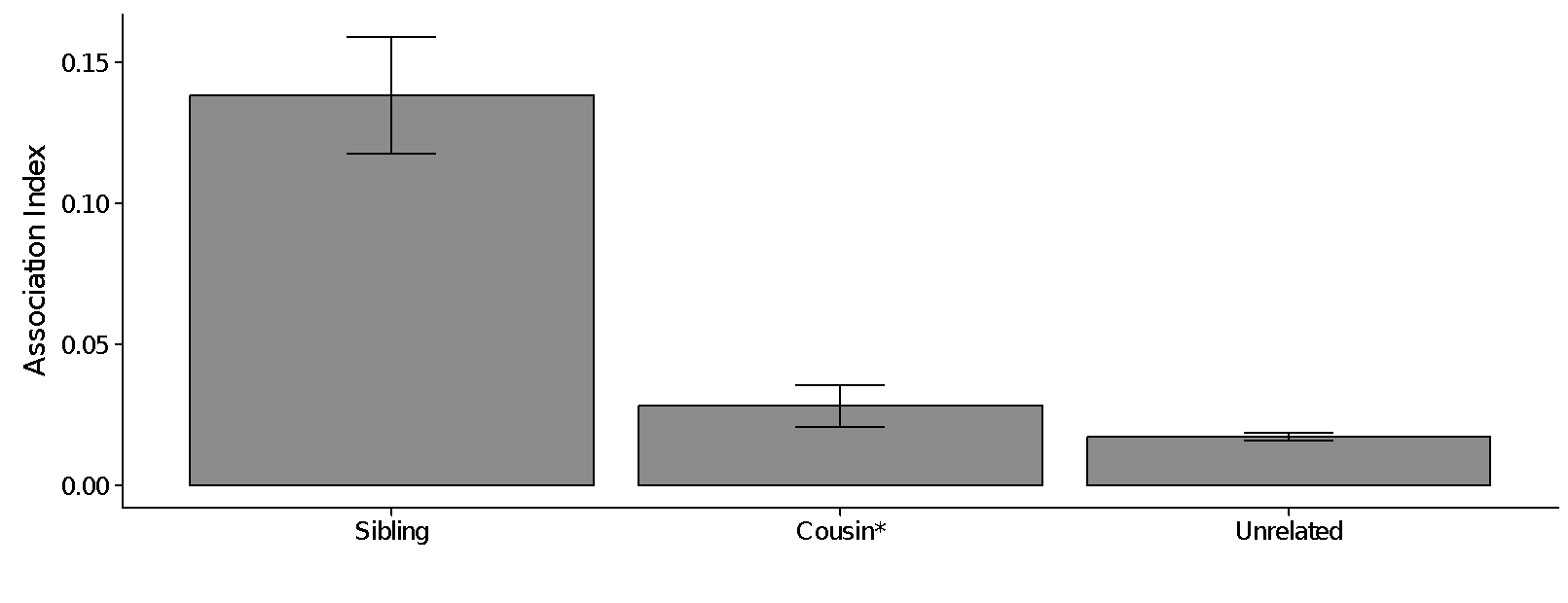
**

**Figure S3. Newman’s modularity across observation periods.** This figure shows modularity as defined by Newman (2004, 2006) to measure the strength of subdivision for networks across each observation period (x-axis: 1 - 6). Modularity was highest in February 2015 (Observation Period 5 on x-axis), which may be the result of clustering of female African buffalo at the end of the reproductive season.


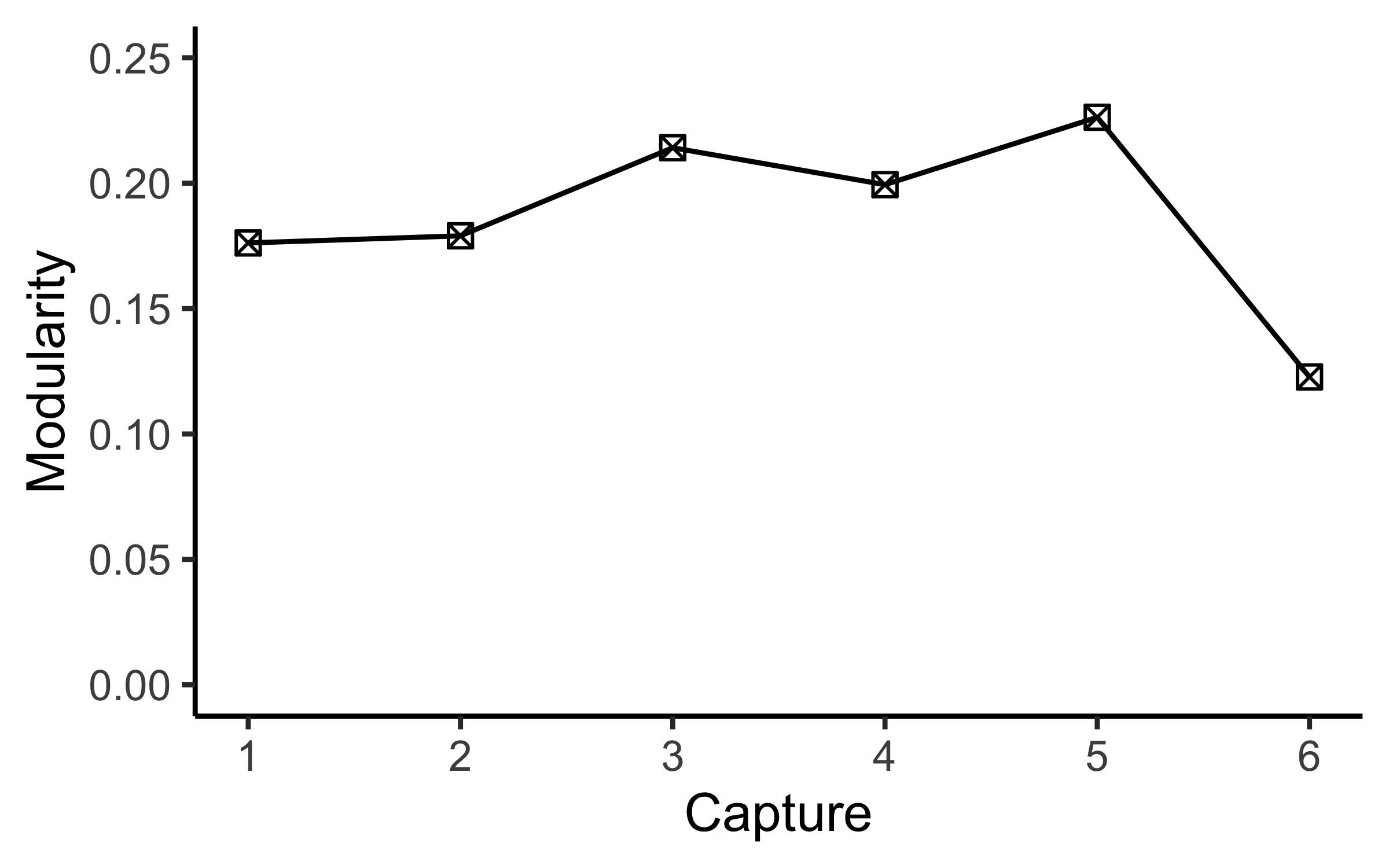


**References**

Boyland, N., James, R., Mlynski, D., Madden, J. & Croft, D. (2013) Spatial proximity loggers for recording animal social networks: consequences of inter-logger variation in performance. *Behavioral Ecology and Sociobiology,* **67,** 1877-1890.

Charleston, B., Bankowski, B.M., Gubbins, S., Chase-Topping, M.E., Schley, D., Howey, R., Barnett, P.V., Gibson, D., Juleff, N.D. & Woolhouse, M.E. (2011) Relationship between clinical signs and transmission of an infectious disease and the implications for control. *Science,* **332,** 726-729.

Grissett, G., White, B. & Larson, R. (2015) Structured literature review of responses of cattle to viral and bacterial pathogens causing bovine respiratory disease complex. *Journal of Veterinary Internal Medicine,* **29,** 770-780.

Jones, O.R. & Wang, J. (2010) COLONY: a program for parentage and sibship inference from multilocus genotype data. *Molecular Ecology Resources,* **10,** 551-555.

Lynch, M. & Ritland, K. (1999) Estimation of pairwise relatedness with molecular markers. *Genetics,* **152,** 1753-1766.

Milligan, B.G. (2003) Maximum-likelihood estimation of relatedness. *Genetics,* **163,** 1153-1167.

Newman, M.E. and Girvan, M., 2004. Finding and evaluating community structure in networks. *Physical review E*, **69**, p.026113.

Newman, M.E., 2006. Modularity and community structure in networks. *Proceedings of the national academy of sciences*, **103**. 8577-8582.

Pew, J., Muir, P.H., Wang, J. & Frasier, T.R. (2015) related: an R package for analysing pairwise relatedness from codominant molecular markers. *Molecular Ecology Resources,* **15,** 557-561.

Queller, D.C. & Goodnight, K.F. (1989) Estimating Relatedness Using Genetic-Markers. *Evolution,* **43,** 258-275.

Rumble, S.M., Lacroute, P., Dalca, A.V., Fiume, M., Sidow, A. & Brudno, M. (2009) SHRiMP: Accurate Mapping of Short Color-space Reads. *PLoS Computational Biology,* **5,** e1000386.

Wang, S., Meyer, E., McKay, J.K. & Matz, M.V. (2012) 2b-RAD: a simple and flexible method for genome-wide genotyping. *Nature Methods,* **9,** 808.
